# Hexakis(1*H*-imidazole-κ*N* ^3^)nickel(II) triaqua­tris(1*H*-imidazole-κ*N* ^3^)nickel(II) bis­(naphthalene-1,4-dicarboxyl­ate)

**DOI:** 10.1107/S1600536808024215

**Published:** 2008-08-06

**Authors:** Jun-Hua Li, Jing-Jing Nie, Duan-Jun Xu

**Affiliations:** a Department of Chemistry, Zhejiang University, People’s Republic of China

## Abstract

The crystal structure of the title compound, [Ni(C_3_H_4_N_2_)_6_][Ni(C_3_H_4_N_2_)_3_(H_2_O)_3_](C_12_H_6_O_4_)_2_, contains uncoordinated naphthalene­dicarboxyl­ate dianions and two kinds of Ni^II^ complex cations, both assuming distorted octa­hedral geometries. One Ni^II^ ion is located on an inversion center and is coordinated by six imidazole mol­ecules, while the other Ni^II^ ion is located on a twofold rotation axis and is coordinated by three water mol­ecules and three imidazole mol­ecules in a *mer*-NiN_3_O_3_ arrangement. The naphthalene­dicarboxyl­ate dianion links both Ni^II^ complex cations *via* O—H⋯O and N—H⋯O hydrogen bonding, but no π–π stacking is observed between aromatic rings in the crystal structure. One imidazole ligand is equally disordered over two sites about a twofold rotation axis; one N atom and one water O atom have site symmetry 2.

## Related literature

For general background, see: Su & Xu (2004 ▶); Xu *et al.* (2007 ▶). For related structures, see: Derissen *et al.* (1979 ▶); Li *et al.* (2008 ▶).
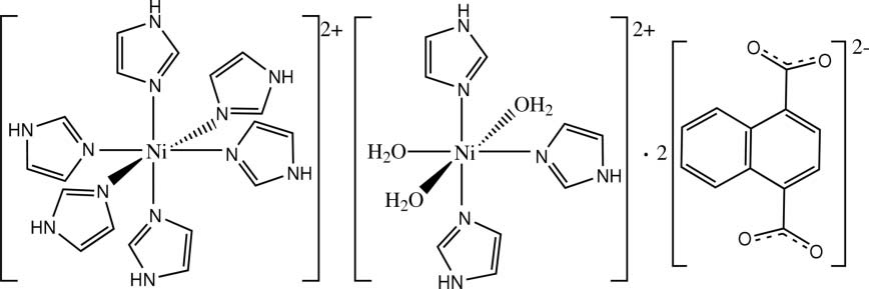


## Experimental

### 

#### Crystal data

[Ni(C_3_H_4_N_2_)_6_][Ni(C_3_H_4_N_2_)_3_(H_2_O)_3_](C_12_H_6_O_4_)_2_
*M* *_r_* = 1212.54
Orthorhombic, 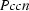

*a* = 29.301 (7) Å
*b* = 9.297 (2) Å
*c* = 20.381 (5) Å
*V* = 5552 (2) Å^3^
*Z* = 4
Mo *K*α radiation
μ = 0.75 mm^−1^
*T* = 294 (2) K
0.22 × 0.15 × 0.10 mm

#### Data collection

Rigaku R-AXIS RAPID IP diffractometer
Absorption correction: multi-scan (*ABSCOR*; Higashi, 1995 ▶) *T*_min_ = 0.866, *T*_max_ = 0.925
33285 measured reflections
4984 independent reflections
2653 reflections with *I* > 2σ(*I*)
*R*_int_ = 0.128

#### Refinement

*R*[*F* ^2^ > 2σ(*F* ^2^)] = 0.056
*wR*(*F* ^2^) = 0.140
*S* = 1.01
4984 reflections
367 parameters
5 restraints
H-atom parameters constrained
Δρ_max_ = 0.95 e Å^−3^
Δρ_min_ = −0.47 e Å^−3^

### 

Data collection: *PROCESS-AUTO* (Rigaku, 1998 ▶); cell refinement: *PROCESS-AUTO*; data reduction: *CrystalStructure* (Rigaku/MSC, 2002 ▶); program(s) used to solve structure: *SIR92* (Altomare *et al.*, 1993 ▶); program(s) used to refine structure: *SHELXL97* (Sheldrick, 2008 ▶); molecular graphics: *ORTEP-3 for Windows* (Farrugia, 1997 ▶); software used to prepare material for publication: *WinGX* (Farrugia, 1999 ▶).

## Supplementary Material

Crystal structure: contains datablocks I, global. DOI: 10.1107/S1600536808024215/hb2767sup1.cif

Structure factors: contains datablocks I. DOI: 10.1107/S1600536808024215/hb2767Isup2.hkl

Additional supplementary materials:  crystallographic information; 3D view; checkCIF report

## Figures and Tables

**Table 1:** Selected bond lengths (Å)

Ni1—N1	2.104 (3)
Ni1—N3	2.120 (4)
Ni1—N5	2.128 (4)
Ni2—O1*W*	2.140 (3)
Ni2—O2*W*	2.025 (4)
Ni2—N7	2.108 (4)
Ni2—N9	2.048 (5)

**Table 2:** Hydrogen-bond geometry (Å, °)

*D*—H⋯*A*	*D*—H	H⋯*A*	*D*⋯*A*	*D*—H⋯*A*
O1*W*—H1*A*⋯O1	0.92	1.87	2.779 (4)	167
O1*W*—H1*B*⋯O3^i^	0.85	2.04	2.884 (4)	172
O2*W*—H2*A*⋯O2	0.84	1.80	2.633 (5)	170
N2—H2*N*⋯O1	0.86	1.87	2.724 (5)	174
N4—H4*N*⋯O4^ii^	0.86	1.90	2.759 (5)	177
N6—H6*N*⋯O4^iii^	0.86	1.98	2.834 (5)	177
N8—H8*N*⋯O3^iv^	0.86	2.04	2.876 (5)	165
N10—H10*A*⋯O2^v^	0.86	1.87	2.638 (8)	149

Symmetry codes: (i) 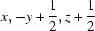; (ii) 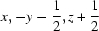; (iii) 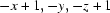; (iv) 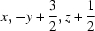; (v) 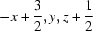.

**Fig. 1.**
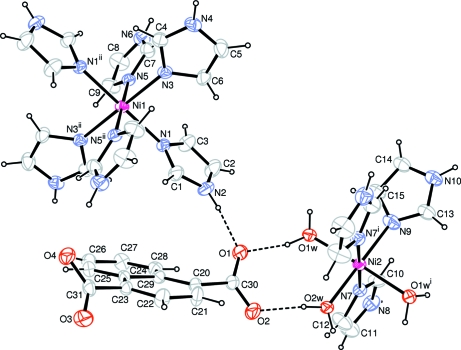
The molecular structure of (I) with 30% probability displacement (arbitrary spheres for H atoms). One of the disordered imidazole components has been omitted for clarify. Dashed lines indicate hydrogen bonding [symmetry codes: (i) -x + 3/2, -y + 3/2, z; (ii) -x + 3/2, -y + 1/2, z + 1].

## supplementary crystallographic information

### Comment

As part of our ongoing investigation on the nature of π-π stacking (Su & Xu, 2004; Xu *et al.*, 2007), the title compound, (I), incorporating naphthalenedicarboxylate dianions, has recently been prepared in the laboratory and its crystal structure is reported here.

The crystal structure contains uncoordinated naphthalenedicarboxylate dianions and two independent Ni^II^ complex cations (Fig. 1). Both Ni^II^ complexes assume distorted octahedral geometry. The Ni1 atom is located in an inversion center and coordinated by six imidazole ligands, while the Ni2 atom is located on a twofold axis and coordinated by three water and three imidazole ligands. In the Ni2-containing complex cation, the O2W and N9 atoms are also located on the twofold axis, but the other atoms of the disordered N9-imidazole ring do not lie on the twofold axis and the N9-imidazole ring is tilted to the twofold axis by an angle of 11.9 (5)°, similar to 14.2 (3)° found in the Mn^II^ analogue (Li *et al.*, 2008). The coordination bond distances (Table 1) are significantly shorter than those found in the Mn^II^ analogue (Li *et al.*, 2008).

The uncoordinated naphthalenedicarboxylate dianion links with both Ni^II^ complex cations *via* O—H···O and N—H···O hydrogen bonding (Fig. 1 and Table 2). Two carboxyl groups are twisted with respect to the naphthalene ring system by dihedral angles of 56.4 (5)° and 50.4 (5)°, which are larger than those found in the structure of free naphthalenedicarboxylic acid (*ca* 40°; Derissen *et al.*, 1979). No π-π stacking is observed between aromatic rings in the crystal structure.

### Experimental

A water-ethanol solution (16 ml, 1:3 v/v) of naphthalene-1,4-dicarboxyllic acid (0.108 g, 0.5 mmol) and sodium carbonate (0.053 g, 0.5 mmol) was refluxed for 0.5 h, then nickel chloride hexahydrate (0.118 g, 0.5 mmol) was added to the above solution. The reaction mixture was refluxed for a further 6.5 h, then imidazole (0.102 g, 1.5 mmol) was added to the above solution and the reaction mixture was refluxed for another 0.5 h. After cooling to room temperature the solution was filtered.  Green prisms of (I) were obtained from the filtrate after 4 d.

### Refinement

The N9-containing imidazole molecule is disordered over two sites, close to a twofold rotation axis, but N9 atom is located on the twofold axis and is not disordered. The disordered components were refined with a half site occupancy and bond-length restraints were used to stabilise the refinement.

The water H atoms were located in a difference Fourier map and refined as riding in as-found relative positions with *U*_iso_(H) = 1.5*U*_eq_(O). Other H atoms were placed in calculated positions with C—H = 0.93 Å and N—H = 0.86 Å, and refined in riding mode with *U*_iso_(H) = 1.2*U*_eq_(C,*N*). The highest peak in the final difference Fourier map is 0.10 Å from N9.

### Crystal data

**Table:**

[Ni(C_3_H_4_N_2_)_6_][Ni(C_3_H_4_N_2_)_3_(H_2_O)_3_](C_12_H_6_O_4_)_2_	*F*_000_ = 2520
*M**_r_* = 1212.54	*D*_x_ = 1.451 Mg m^−^^3^
Orthorhombic, *P**c**c**n*	Mo *K*α radiation λ = 0.71073 Å
Hall symbol: -P 2ab 2ac	Cell parameters from 5668 reflections
*a* = 29.301 (7) Å	θ = 2.2–24.5º
*b* = 9.297 (2) Å	µ = 0.75 mm^−^^1^
*c* = 20.381 (5) Å	*T* = 294 (2) K
*V* = 5552 (2) Å^3^	Prism, green
*Z* = 4	0.22 × 0.15 × 0.10 mm

### Data collection

**Table:**

Rigaku R-AXIS RAPID IP diffractometer	4984 independent reflections
Radiation source: fine-focus sealed tube	2653 reflections with *I* > 2σ(*I*)
Monochromator: graphite	*R*_int_ = 0.128
Detector resolution: 10.0 pixels mm^-1^	θ_max_ = 25.2º
*T* = 294(2) K	θ_min_ = 1.4º
ω scans	*h* = −34→34
Absorption correction: multi-scan(ABSCOR; Higashi, 1995)	*k* = −9→11
*T*_min_ = 0.866, *T*_max_ = 0.925	*l* = −24→23
33285 measured reflections	

### Refinement

**Table:**

Refinement on *F*^2^	Secondary atom site location: difference Fourier map
Least-squares matrix: full	Hydrogen site location: inferred from neighbouring sites
*R*[*F*^2^ > 2σ(*F*^2^)] = 0.056	H-atom parameters constrained
*wR*(*F*^2^) = 0.140	*w* = 1/[σ^2^(*F*_o_^2^) + (0.0559*P*)^2^] where *P* = (*F*_o_^2^ + 2*F*_c_^2^)/3
*S* = 1.01	(Δ/σ)_max_ < 0.001
4984 reflections	Δρ_max_ = 0.95 e Å^−^^3^
367 parameters	Δρ_min_ = −0.47 e Å^−^^3^
5 restraints	Extinction correction: none
Primary atom site location: structure-invariant direct methods	

### Special details

**Table:**

Geometry. All e.s.d.'s (except the e.s.d. in the dihedral angle between two l.s. planes) are estimated using the full covariance matrix. The cell e.s.d.'s are taken into account individually in the estimation of e.s.d.'s in distances, angles and torsion angles; correlations between e.s.d.'s in cell parameters are only used when they are defined by crystal symmetry. An approximate (isotropic) treatment of cell e.s.d.'s is used for estimating e.s.d.'s involving l.s. planes.
Refinement. Refinement of *F*^2^ against ALL reflections. The weighted *R*-factor *wR* and goodness of fit *S* are based on *F*^2^, conventional *R*-factors *R* are based on *F*, with *F* set to zero for negative *F*^2^. The threshold expression of *F*^2^ > σ(*F*^2^) is used only for calculating *R*-factors(gt) *etc*. and is not relevant to the choice of reflections for refinement. *R*-factors based on *F*^2^ are statistically about twice as large as those based on *F*, and *R*- factors based on ALL data will be even larger.

### Fractional atomic coordinates and isotropic or equivalent isotropic displacement parameters (Å^2^)

**Table:**

	*x*	*y*	*z*	*U*_iso_*/*U*_eq_	Occ. (<1)
Ni1	0.5000	0.0000	0.5000	0.0358 (2)	
Ni2	0.7500	0.7500	0.54570 (4)	0.0380 (2)	
N1	0.53093 (12)	0.2040 (4)	0.50465 (18)	0.0392 (9)	
N2	0.58307 (14)	0.3703 (4)	0.4922 (2)	0.0586 (12)	
H2N	0.6058	0.4167	0.4759	0.070*	
N3	0.54557 (13)	−0.0704 (4)	0.57414 (17)	0.0417 (10)	
N4	0.58361 (13)	−0.2193 (4)	0.63876 (19)	0.0505 (11)	
H4N	0.5921	−0.2980	0.6573	0.061*	
N5	0.45162 (13)	0.0630 (4)	0.57239 (18)	0.0399 (9)	
N6	0.41513 (15)	0.0694 (4)	0.6666 (2)	0.0570 (12)	
H6N	0.4091	0.0576	0.7075	0.068*	
N7	0.72172 (12)	0.9585 (4)	0.54389 (19)	0.0449 (10)	
N8	0.69577 (14)	1.1727 (4)	0.5723 (2)	0.0612 (12)	
H8N	0.6878	1.2441	0.5966	0.073*	
O1	0.65246 (12)	0.5137 (4)	0.43190 (15)	0.0622 (10)	
O2	0.69666 (14)	0.6205 (4)	0.36079 (17)	0.0887 (14)	
O3	0.65981 (12)	0.0720 (3)	0.13293 (16)	0.0598 (10)	
O4	0.60816 (11)	−0.0274 (3)	0.19982 (15)	0.0556 (9)	
O1W	0.68332 (10)	0.6562 (3)	0.54323 (13)	0.0447 (8)	
H1A	0.6741	0.6217	0.5028	0.067*	
H1B	0.6780	0.5935	0.5724	0.067*	
O2W	0.7500	0.7500	0.44632 (18)	0.0498 (12)	
H2A	0.7342	0.6989	0.4207	0.075*	
C1	0.56657 (18)	0.2458 (6)	0.4712 (2)	0.0546 (14)	
H1	0.5790	0.1939	0.4365	0.066*	
C2	0.5574 (2)	0.4100 (6)	0.5438 (3)	0.0690 (17)	
H2	0.5610	0.4913	0.5698	0.083*	
C3	0.52539 (18)	0.3080 (5)	0.5499 (3)	0.0598 (15)	
H3	0.5023	0.3089	0.5812	0.072*	
C4	0.54989 (17)	−0.2028 (5)	0.5955 (2)	0.0494 (13)	
H4	0.5312	−0.2780	0.5818	0.059*	
C5	0.60167 (18)	−0.0877 (6)	0.6475 (3)	0.0652 (16)	
H5	0.6256	−0.0637	0.6755	0.078*	
C6	0.57841 (17)	0.0030 (5)	0.6078 (2)	0.0547 (14)	
H6	0.5840	0.1011	0.6040	0.066*	
C7	0.45366 (17)	0.0319 (5)	0.6357 (2)	0.0501 (13)	
H7	0.4786	−0.0106	0.6561	0.060*	
C8	0.38765 (19)	0.1290 (6)	0.6211 (3)	0.0700 (17)	
H8	0.3587	0.1667	0.6282	0.084*	
C9	0.40963 (19)	0.1237 (6)	0.5646 (3)	0.0635 (15)	
H9	0.3980	0.1568	0.5248	0.076*	
C10	0.71259 (18)	1.0466 (5)	0.5928 (3)	0.0603 (15)	
H10	0.7173	1.0236	0.6367	0.072*	
C11	0.6937 (2)	1.1662 (6)	0.5064 (3)	0.0779 (18)	
H11	0.6832	1.2378	0.4783	0.093*	
C12	0.7098 (2)	1.0357 (6)	0.4888 (3)	0.0743 (18)	
H12	0.7124	1.0028	0.4459	0.089*	
C20	0.65761 (16)	0.4155 (5)	0.3246 (2)	0.0409 (12)	
C21	0.69215 (17)	0.3443 (5)	0.2948 (2)	0.0571 (14)	
H21	0.7221	0.3675	0.3057	0.069*	
C22	0.68420 (16)	0.2359 (5)	0.2477 (2)	0.0541 (14)	
H22	0.7090	0.1911	0.2278	0.065*	
C23	0.64133 (15)	0.1953 (5)	0.2307 (2)	0.0395 (11)	
C24	0.60336 (14)	0.2706 (5)	0.25953 (19)	0.0360 (11)	
C25	0.55749 (15)	0.2431 (5)	0.2410 (2)	0.0443 (12)	
H25	0.5514	0.1709	0.2107	0.053*	
C26	0.52247 (17)	0.3195 (5)	0.2666 (2)	0.0511 (13)	
H26	0.4927	0.2974	0.2544	0.061*	
C27	0.53041 (17)	0.4317 (5)	0.3111 (2)	0.0525 (14)	
H27	0.5061	0.4849	0.3276	0.063*	
C28	0.57430 (17)	0.4625 (5)	0.3302 (2)	0.0493 (13)	
H28	0.5794	0.5366	0.3599	0.059*	
C29	0.61186 (15)	0.3837 (4)	0.3056 (2)	0.0376 (11)	
C30	0.66958 (17)	0.5248 (5)	0.3766 (2)	0.0478 (13)	
C31	0.63586 (17)	0.0716 (5)	0.1832 (2)	0.0447 (12)	
N9	0.7500	0.7500	0.6462 (3)	0.0595 (12)	
N10	0.7702 (3)	0.7153 (9)	0.7485 (3)	0.0595 (12)	0.50
H10A	0.7872	0.7128	0.7829	0.071*	0.50
C13	0.7844 (2)	0.7501 (13)	0.6881 (3)	0.0595 (12)	0.50
H13	0.8144	0.7715	0.6771	0.071*	0.50
C14	0.7248 (3)	0.6846 (11)	0.7474 (4)	0.0595 (12)	0.50
H14	0.7062	0.6550	0.7818	0.071*	0.50
C15	0.7135 (2)	0.7078 (13)	0.6836 (4)	0.0595 (12)	0.50
H15	0.6841	0.6963	0.6671	0.071*	0.50

### Atomic displacement parameters (Å^2^)

**Table:**

	*U*^11^	*U*^22^	*U*^33^	*U*^12^	*U*^13^	*U*^23^
Ni1	0.0416 (5)	0.0306 (5)	0.0353 (4)	0.0021 (4)	0.0028 (4)	0.0023 (4)
Ni2	0.0463 (5)	0.0360 (5)	0.0316 (5)	−0.0068 (4)	0.000	0.000
N1	0.042 (2)	0.033 (2)	0.043 (2)	−0.0010 (18)	−0.002 (2)	0.0025 (19)
N2	0.065 (3)	0.048 (3)	0.063 (3)	−0.020 (2)	0.006 (2)	0.006 (2)
N3	0.052 (3)	0.031 (2)	0.042 (2)	0.001 (2)	0.001 (2)	0.0034 (19)
N4	0.053 (3)	0.046 (3)	0.052 (3)	0.010 (2)	−0.007 (2)	0.012 (2)
N5	0.045 (3)	0.033 (2)	0.041 (2)	−0.0024 (19)	0.0020 (19)	0.0027 (18)
N6	0.063 (3)	0.064 (3)	0.044 (3)	−0.002 (2)	0.016 (2)	−0.004 (2)
N7	0.048 (2)	0.040 (2)	0.046 (3)	−0.0051 (19)	−0.002 (2)	0.001 (2)
N8	0.062 (3)	0.039 (3)	0.082 (4)	0.001 (2)	0.006 (3)	−0.002 (3)
O1	0.084 (3)	0.066 (3)	0.0359 (19)	−0.031 (2)	0.0086 (19)	−0.0142 (18)
O2	0.118 (3)	0.101 (3)	0.047 (2)	−0.069 (3)	0.004 (2)	−0.013 (2)
O3	0.084 (3)	0.052 (2)	0.043 (2)	−0.0058 (19)	0.022 (2)	−0.0143 (17)
O4	0.070 (2)	0.045 (2)	0.052 (2)	−0.0141 (18)	0.0050 (18)	−0.0095 (17)
O1W	0.053 (2)	0.047 (2)	0.0348 (18)	−0.0090 (15)	0.0016 (15)	0.0027 (15)
O2W	0.068 (3)	0.054 (3)	0.028 (2)	−0.027 (2)	0.000	0.000
C1	0.069 (4)	0.046 (3)	0.048 (3)	−0.013 (3)	0.009 (3)	−0.010 (3)
C2	0.083 (4)	0.043 (3)	0.081 (5)	−0.013 (3)	0.004 (4)	−0.018 (3)
C3	0.059 (4)	0.041 (3)	0.080 (4)	0.000 (3)	0.015 (3)	−0.011 (3)
C4	0.053 (3)	0.043 (3)	0.053 (3)	0.003 (2)	−0.007 (3)	0.007 (3)
C5	0.069 (4)	0.051 (4)	0.076 (4)	0.004 (3)	−0.032 (3)	0.001 (3)
C6	0.060 (3)	0.032 (3)	0.073 (4)	−0.003 (3)	−0.019 (3)	0.004 (3)
C7	0.054 (3)	0.057 (3)	0.039 (3)	0.001 (3)	0.004 (3)	−0.007 (3)
C8	0.059 (4)	0.084 (5)	0.067 (4)	0.028 (3)	0.022 (3)	−0.003 (3)
C9	0.062 (4)	0.070 (4)	0.058 (4)	0.018 (3)	0.003 (3)	0.010 (3)
C10	0.085 (4)	0.039 (3)	0.057 (4)	0.000 (3)	0.005 (3)	0.007 (3)
C11	0.093 (5)	0.064 (4)	0.077 (5)	0.026 (3)	−0.008 (4)	0.013 (4)
C12	0.106 (5)	0.057 (4)	0.060 (4)	0.029 (3)	−0.012 (3)	0.007 (3)
C20	0.049 (3)	0.038 (3)	0.037 (3)	−0.007 (2)	0.000 (2)	−0.007 (2)
C21	0.043 (3)	0.070 (4)	0.058 (3)	−0.015 (3)	−0.003 (3)	−0.023 (3)
C22	0.041 (3)	0.062 (4)	0.059 (3)	−0.002 (3)	0.008 (3)	−0.018 (3)
C23	0.040 (3)	0.046 (3)	0.033 (3)	−0.005 (2)	0.001 (2)	−0.007 (2)
C24	0.038 (3)	0.041 (3)	0.029 (2)	−0.001 (2)	−0.001 (2)	−0.001 (2)
C25	0.044 (3)	0.048 (3)	0.041 (3)	−0.003 (3)	−0.007 (2)	−0.004 (2)
C26	0.042 (3)	0.060 (3)	0.052 (3)	−0.001 (3)	−0.004 (3)	0.003 (3)
C27	0.048 (3)	0.050 (3)	0.059 (4)	0.019 (3)	−0.003 (3)	−0.003 (3)
C28	0.057 (3)	0.048 (3)	0.043 (3)	0.004 (3)	0.001 (3)	−0.002 (2)
C29	0.043 (3)	0.034 (3)	0.036 (3)	0.001 (2)	−0.002 (2)	0.000 (2)
C30	0.056 (3)	0.048 (3)	0.040 (3)	−0.013 (3)	0.000 (3)	−0.008 (2)
C31	0.057 (3)	0.037 (3)	0.040 (3)	0.001 (3)	−0.011 (3)	−0.010 (2)
N9	0.078 (3)	0.062 (3)	0.039 (2)	0.005 (3)	0.000	0.000
N10	0.078 (3)	0.062 (3)	0.039 (2)	0.005 (3)	0.000	0.000
C13	0.078 (3)	0.062 (3)	0.039 (2)	0.005 (3)	0.000	0.000
C14	0.078 (3)	0.062 (3)	0.039 (2)	0.005 (3)	0.000	0.000
C15	0.078 (3)	0.062 (3)	0.039 (2)	0.005 (3)	0.000	0.000

### Geometric parameters (Å, °)

**Table:**

Ni1—N1	2.104 (3)	C4—H4	0.9300
Ni1—N1^i^	2.104 (3)	C5—C6	1.353 (6)
Ni1—N3	2.120 (4)	C5—H5	0.9300
Ni1—N3^i^	2.120 (4)	C6—H6	0.9300
Ni1—N5	2.128 (4)	C7—H7	0.9300
Ni1—N5^i^	2.128 (4)	C8—C9	1.321 (6)
Ni2—O1W^ii^	2.140 (3)	C8—H8	0.9300
Ni2—O1W	2.140 (3)	C9—H9	0.9300
Ni2—O2W	2.025 (4)	C10—H10	0.9300
Ni2—N7^ii^	2.108 (4)	C11—C12	1.349 (7)
Ni2—N7	2.108 (4)	C11—H11	0.9300
Ni2—N9^ii^	2.048 (5)	C12—H12	0.9300
Ni2—N9	2.048 (5)	C20—C21	1.353 (6)
N1—C1	1.306 (5)	C20—C29	1.426 (6)
N1—C3	1.346 (6)	C20—C30	1.510 (6)
N2—C1	1.325 (6)	C21—C22	1.411 (6)
N2—C2	1.345 (6)	C21—H21	0.9300
N2—H2N	0.8600	C22—C23	1.357 (6)
N3—C4	1.312 (5)	C22—H22	0.9300
N3—C6	1.365 (5)	C23—C24	1.440 (6)
N4—C4	1.333 (5)	C23—C31	1.511 (6)
N4—C5	1.344 (6)	C24—C25	1.419 (6)
N4—H4N	0.8600	C24—C29	1.432 (5)
N5—C7	1.323 (5)	C25—C26	1.352 (6)
N5—C9	1.363 (6)	C25—H25	0.9300
N6—C7	1.339 (5)	C26—C27	1.402 (6)
N6—C8	1.347 (6)	C26—H26	0.9300
N6—H6N	0.8600	C27—C28	1.374 (6)
N7—C10	1.317 (6)	C27—H27	0.9300
N7—C12	1.377 (6)	C28—C29	1.414 (6)
N8—C10	1.338 (6)	C28—H28	0.9300
N8—C11	1.347 (6)	N9—N9^ii^	0.000 (10)
N8—H8N	0.8600	N9—C13	1.3201 (11)
O1—C30	1.238 (5)	N9—C13^ii^	1.3201 (11)
O2—C30	1.235 (5)	N9—C15^ii^	1.3703 (11)
O3—C31	1.243 (5)	N9—C15	1.3703 (11)
O4—C31	1.272 (5)	N10—C13	1.3399 (11)
O1W—H1A	0.9247	N10—C14	1.3603 (11)
O1W—H1B	0.8470	N10—H10A	0.8600
O2W—H2A	0.8443	C13—H13	0.9300
C1—H1	0.9300	C14—C15	1.3599 (11)
C2—C3	1.338 (7)	C14—C14^ii^	1.912 (16)
C2—H2	0.9300	C14—H14	0.9300
C3—H3	0.9300	C15—H15	0.9300
			
N1—Ni1—N1^i^	180.0	C5—C6—H6	124.9
N1—Ni1—N3	88.56 (14)	N3—C6—H6	124.9
N1^i^—Ni1—N3	91.44 (14)	N5—C7—N6	111.3 (4)
N1—Ni1—N3^i^	91.44 (14)	N5—C7—H7	124.3
N1^i^—Ni1—N3^i^	88.56 (14)	N6—C7—H7	124.3
N3—Ni1—N3^i^	180.0	C9—C8—N6	107.1 (5)
N1—Ni1—N5	90.44 (14)	C9—C8—H8	126.5
N1^i^—Ni1—N5	89.56 (14)	N6—C8—H8	126.5
N3—Ni1—N5	90.59 (14)	C8—C9—N5	110.7 (5)
N3^i^—Ni1—N5	89.41 (14)	C8—C9—H9	124.7
N1—Ni1—N5^i^	89.56 (14)	N5—C9—H9	124.7
N1^i^—Ni1—N5^i^	90.44 (14)	N7—C10—N8	112.6 (5)
N3—Ni1—N5^i^	89.41 (14)	N7—C10—H10	123.7
N3^i^—Ni1—N5^i^	90.59 (14)	N8—C10—H10	123.7
N5—Ni1—N5^i^	180.0	N8—C11—C12	106.8 (5)
O2W—Ni2—N9	180.0	N8—C11—H11	126.6
O2W—Ni2—N7^ii^	89.00 (11)	C12—C11—H11	126.6
N9^ii^—Ni2—N7^ii^	91.00 (11)	C11—C12—N7	109.9 (5)
O2W—Ni2—N7	89.00 (11)	C11—C12—H12	125.0
N9—Ni2—N7	91.00 (11)	N7—C12—H12	125.0
N7^ii^—Ni2—N7	178.0 (2)	C21—C20—C29	118.7 (4)
O2W—Ni2—O1W^ii^	88.65 (8)	C21—C20—C30	118.1 (4)
N9—Ni2—O1W^ii^	91.35 (8)	C29—C20—C30	123.2 (4)
N7^ii^—Ni2—O1W^ii^	90.87 (12)	C20—C21—C22	122.1 (4)
N7—Ni2—O1W^ii^	89.08 (12)	C20—C21—H21	119.0
O2W—Ni2—O1W	88.65 (8)	C22—C21—H21	119.0
N9—Ni2—O1W	91.35 (8)	C23—C22—C21	121.7 (4)
N7^ii^—Ni2—O1W	89.08 (12)	C23—C22—H22	119.2
N7—Ni2—O1W	90.87 (12)	C21—C22—H22	119.2
O1W^ii^—Ni2—O1W	177.31 (15)	C22—C23—C24	118.4 (4)
C1—N1—C3	103.9 (4)	C22—C23—C31	118.3 (4)
C1—N1—Ni1	126.1 (3)	C24—C23—C31	123.3 (4)
C3—N1—Ni1	128.8 (3)	C25—C24—C29	118.1 (4)
C1—N2—C2	106.7 (4)	C25—C24—C23	122.4 (4)
C1—N2—H2N	126.7	C29—C24—C23	119.4 (4)
C2—N2—H2N	126.7	C26—C25—C24	121.5 (4)
C4—N3—C6	103.5 (4)	C26—C25—H25	119.3
C4—N3—Ni1	126.0 (3)	C24—C25—H25	119.3
C6—N3—Ni1	130.4 (3)	C25—C26—C27	120.9 (5)
C4—N4—C5	106.0 (4)	C25—C26—H26	119.5
C4—N4—H4N	127.0	C27—C26—H26	119.5
C5—N4—H4N	127.0	C28—C27—C26	119.6 (5)
C7—N5—C9	104.2 (4)	C28—C27—H27	120.2
C7—N5—Ni1	125.8 (3)	C26—C27—H27	120.2
C9—N5—Ni1	129.3 (3)	C27—C28—C29	121.4 (4)
C7—N6—C8	106.7 (4)	C27—C28—H28	119.3
C7—N6—H6N	126.6	C29—C28—H28	119.3
C8—N6—H6N	126.6	C28—C29—C20	121.9 (4)
C10—N7—C12	103.9 (4)	C28—C29—C24	118.5 (4)
C10—N7—Ni2	129.7 (3)	C20—C29—C24	119.5 (4)
C12—N7—Ni2	126.4 (3)	O2—C30—O1	123.9 (4)
C10—N8—C11	106.7 (5)	O2—C30—C20	116.8 (4)
C10—N8—H8N	126.6	O1—C30—C20	119.3 (4)
C11—N8—H8N	126.6	O3—C31—O4	125.6 (4)
Ni2—O1W—H1A	115.3	O3—C31—C23	117.7 (4)
Ni2—O1W—H1B	115.5	O4—C31—C23	116.6 (4)
H1A—O1W—H1B	109.4	C13—N9—C15	103.6 (6)
Ni2—O2W—H2A	128.2	C13—N9—Ni2	130.3 (4)
N1—C1—N2	112.6 (4)	C15—N9—Ni2	123.8 (4)
N1—C1—H1	123.7	C13—N10—C14	109.8 (8)
N2—C1—H1	123.7	C13—N10—H10A	125.1
C3—C2—N2	105.7 (5)	C14—N10—H10A	125.1
C3—C2—H2	127.2	N9—C13—N10	111.0 (7)
N2—C2—H2	127.2	N9—C13—H13	124.5
C2—C3—N1	111.2 (5)	N10—C13—H13	124.5
C2—C3—H3	124.4	C15—C14—N10	102.8 (8)
N1—C3—H3	124.4	C15—C14—C14^ii^	95.0 (5)
N3—C4—N4	113.5 (4)	C15—C14—H14	128.6
N3—C4—H4	123.2	N10—C14—H14	128.6
N4—C4—H4	123.2	C14^ii^—C14—H14	129.9
N4—C5—C6	106.8 (5)	C14—C15—N9	112.8 (7)
N4—C5—H5	126.6	C14—C15—H15	123.6
C6—C5—H5	126.6	N9—C15—H15	123.6
C5—C6—N3	110.1 (4)		

Symmetry codes: (i) −*x*+1, −*y*, −*z*+1; (ii) −*x*+3/2, −*y*+3/2, *z*.

### Hydrogen-bond geometry (Å, °)

**Table:**

*D*—H···*A*	*D*—H	H···*A*	*D*···*A*	*D*—H···*A*
O1W—H1A···O1	0.92	1.87	2.779 (4)	167
O1W—H1B···O3^iii^	0.85	2.04	2.884 (4)	172
O2W—H2A···O2	0.84	1.80	2.633 (5)	170
N2—H2N···O1	0.86	1.87	2.724 (5)	174
N4—H4N···O4^iv^	0.86	1.90	2.759 (5)	177
N6—H6N···O4^i^	0.86	1.98	2.834 (5)	177
N8—H8N···O3^v^	0.86	2.04	2.876 (5)	165
N10—H10A···O2^vi^	0.86	1.87	2.638 (8)	149

Symmetry codes: (iii) *x*, −*y*+1/2, *z*+1/2; (iv) *x*, −*y*−1/2, *z*+1/2; (i) −*x*+1, −*y*, −*z*+1; (v) *x*, −*y*+3/2, *z*+1/2; (vi) −*x*+3/2, *y*, *z*+1/2.
